# Discovering Thiamine Transporters as Targets of Chloroquine Using a Novel Functional Genomics Strategy

**DOI:** 10.1371/journal.pgen.1003083

**Published:** 2012-11-29

**Authors:** Zhiwei Huang, Sankaranarayanan Srinivasan, Jianhuai Zhang, Kaifu Chen, Yongxiang Li, Wei Li, Florante A. Quiocho, Xuewen Pan

**Affiliations:** 1 Verna and Marrs McLean Department of Biochemistry and Molecular Biology, Baylor College of Medicine, Houston, Texas, United States of America; 2 Institute of Biological Sciences and Biotechnology, Donghua University, Shanghai, China; 3 Division of Biostatistics, Dan L. Duncan Cancer Center, Baylor College of Medicine, Houston, Texas, United States of America; 4 Department of Molecular and Cellular Biology, Baylor College of Medicine, Houston, Texas, United States of America; 5 Department of Molecular and Human Genetics, Baylor College of Medicine, Houston, Texas, United States of America; 6 Center of Molecular Discovery, Baylor College of Medicine, Houston, Texas, United States of America; Johns Hopkins School of Medicine, United States of America

## Abstract

Chloroquine (CQ) and other quinoline-containing antimalarials are important drugs with many therapeutic benefits as well as adverse effects. However, the molecular targets underlying most such effects are largely unknown. By taking a novel functional genomics strategy, which employs a unique combination of genome-wide drug-gene synthetic lethality (DGSL), gene-gene synthetic lethality (GGSL), and dosage suppression (DS) screens in the model organism *Saccharomyces cerevisiae* and is thus termed SL/DS for simplicity, we found that CQ inhibits the thiamine transporters Thi7, Nrt1, and Thi72 in yeast. We first discovered a *thi3Δ* mutant as hypersensitive to CQ using a genome-wide DGSL analysis. Using genome-wide GGSL and DS screens, we then found that a *thi7Δ* mutation confers severe growth defect in the *thi3Δ* mutant and that *THI7* overexpression suppresses CQ-hypersensitivity of this mutant. We subsequently showed that CQ inhibits the functions of Thi7 and its homologues Nrt1 and Thi72. In particular, the transporter activity of wild-type Thi7 but not a CQ-resistant mutant (Thi7^T287N^) was completely inhibited by the drug. Similar effects were also observed with other quinoline-containing antimalarials. In addition, CQ completely inhibited a human thiamine transporter (SLC19A3) expressed in yeast and significantly inhibited thiamine uptake in cultured human cell lines. Therefore, inhibition of thiamine uptake is a conserved mechanism of action of CQ. This study also demonstrated SL/DS as a uniquely effective methodology for discovering drug targets.

## Author Summary

By using a novel SL/DS methodology in the model organism yeast, we discovered that the antimalarial drug CQ inhibits thiamine transporters and consequently causes thiamine (vitamin B1) deficiency and growth defects. This mechanism of action (MOA) is conserved in human cells and possibly also in other organisms. Given that both thiamine deficiency and treatment with CQ cause retinal, neurological, and cardiovascular disorders in humans, our results suggest that thiamine deficiency might be a root cause of some of CQ's adverse effects, which might be preventable with concomitant dietary thiamine supplementation. Such a MOA by CQ could also be responsible for its therapeutic effects against malarial parasites, which need exogenous thiamine for survival. Such a possibility needs to be investigated before dietary thiamine supplementation can be used to prevent CQ's adverse effects.

## Introduction

Chloroquine (CQ) and other quinoline-containing compounds have been major antimalarial drugs for many decades. They are also effective treatments for systematic lupus erythematosus, rheumatoid arthritis, and many other rheumatic and skin diseases [1]. In recent years, their effects in treating viral, bacterial, and fungal infections and cancer have also been explored [2], [3]. Despite being relatively safe, these drugs can cause severe adverse side effects, including retinopathy, myopathy, cardiopathy, peripheral neuropathy, and others [4], [5], [6]. In many cases, the underlying molecular mechanisms of the therapeutic and deleterious effects are not well understood.

The model organism yeast *Saccharomyces cerevisiae* is an excellent system for discovering conserved targets of bioactive compounds [7]. In this study, we took a novel functional genomics approach in yeast to explore the mechanism(s) of action (MOA) of CQ. By first performing a genome-wide drug-gene synthetic lethality (DGSL) screen, we identified 95 CQ-hypersensitive deletion mutants, including those involved in vacuole functions (e.g., *mon2Δ*, *vma4Δ*, and *vma8Δ*) [8], [9], iron homeostasis (e.g., *fet3Δ*) [10], and thiamine metabolism (e.g., *thi3Δ*) [11]. By centering on the *thi3Δ* mutation, we next performed genome-wide gene-gene synthetic lethality (GGSL) and dosage suppression (DS) screens and discovered the high affinity thiamine transporter Thi7 [12] as a candidate target of CQ. For simplicity, this unique combination of DGSL, GGSL, and DS screens was termed SL/DS. We subsequently showed that CQ inhibits Thi7-related functions, particularly Thi7-dependent uptake of thiamine. We also showed that CQ likely inhibits the low affinity thiamine transporters Nrt1 and Thi72 [13] in yeast. This MOA is also shared by other quinoline-containing antimalarials. Moreover, we demonstrated that CQ completely inactivates a human thiamine transporter (SLC19A3) [14], [15] expressed in yeast cells and significantly inhibited thiamine uptake in HeLa and HT1080 cells, suggesting that such a MOA is conserved across species. This study also demonstrated that SL/DS is an effective strategy for drug target identification, especially for discovering non-essential genes as drug targets.

## Results

### A genome-wide DGSL screen revealed multiple distinct functions affected by CQ

To discover the *in vivo* target(s) of CQ that might mediate its effects in a eukaryote, we first explored haploinsufficiency [16] by screening a yeast genome-wide heterozygous diploid deletion library for hypersensitive mutants. This identified six mutants as CQ-hypersensitive, with the *NEO1/neo1Δ* mutant exhibiting the highest sensitivity (Figure S1). The defect of this mutant was complemented with expressing *NEO1* from a plasmid (Figure S1). *NEO1* encodes an essential aminophospholipid flippase involved in endocytosis and vacuolar biogenesis [17]. It is also required for resistance to other compounds [18]. Possibly, Neo1 is generally involved in regulating the accumulation of many different compounds in vacuoles. It is thus not further explored as a target of CQ in this study.

**Figure 1:**
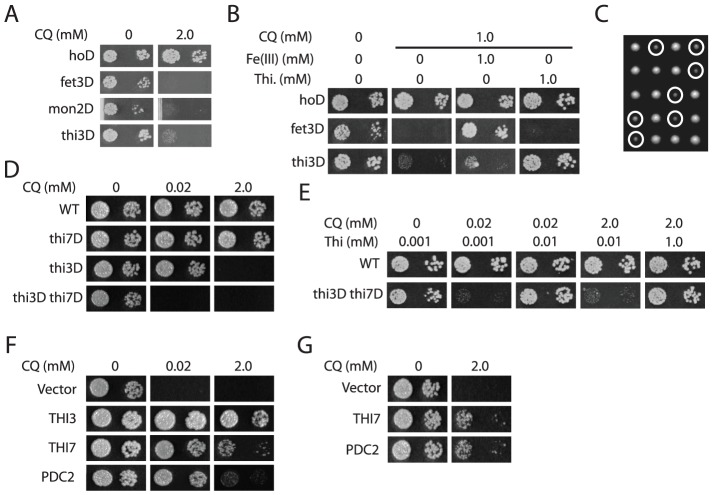
CQ possibly affects multiple biological functions. A. Representative yeast haploid deletion mutants exhibiting the highest levels of sensitivity to CQ. B. Suppression of CQ-hypersensitivity of the *fet3Δ* and *thi3Δ* mutants by exogenously supplied iron [Fe(III)] and thiamine (Thi), respectively. C. Yeast tetrad analysis of the synthetically sick interaction between *thi3Δ* and *thi7Δ*. Colonies of the *thi3Δ thi7Δ* double mutant were all circled. D. Increased CQ-sensitivity of a *thi3Δ thi7Δ* double mutant as compared to either single mutant. E. Dosage-dependent suppression of CQ-hypersensitivity of the *thi3Δ thi7Δ* double mutant by exogenously supplied thiamine. F. Suppression of CQ-hypersensitivity of the *thi3Δ thi7Δ* double mutant by overexpressing *THI3*, *THI7*, and *PDC2*. G. Suppression of CQ-hypersensitivity of the *thi3Δ* single mutant by overexpressing *THI7* and *PDC2*.

With a genome-wide DGSL screen, we next identified and validated 95 CQ-hypersensitive haploid deletion mutants (Table S1). Gene Ontology enrichment analysis revealed that these mostly affected vacuole functions, steroid biosynthesis, endocytosis, iron homeostasis, and post-Golgi transport (Figure S2). However, instead of relying on such an enrichment analysis, we took a novel approach by emphasizing individual mutants exhibiting the highest levels of sensitivity to CQ. We reasoned that such a mutant would most likely affect a function closely related to a drug target. In support of this hypothesis, mutants defective in yeast vacuolar functions (e.g., *vma4Δ* and *mon2Δ*) [8], [9] and iron metabolism (e.g., *fet3Δ*) [10] were among those exhibiting the highest levels of sensitivity (Figure 1A), consistent with the general idea that CQ concentrates in vacuoles or lysosomes [19] and, as previously observed, inhibits iron uptake in budding yeast [20]. In addition, we found that a *thi3Δ* mutant affecting thiamine biosynthesis [11] also exhibited comparably high levels of CQ-hypersensitivity (Figure 1A), and this defect was complemented by expressing *THI3* from a plasmid. Furthermore, a *fet3Δ thi3Δ* double mutant was not apparently more sensitive to CQ than either single mutant (Figure S3), suggesting that the two mutations affect either the same or completely unrelated pathways. Consistent with the latter possibility, CQ-hypersensitive phenotype of the *fet3Δ* mutant was suppressed by exogenously supplied excess amount of iron but not thiamine, whereas that of the *thi3Δ* mutant was suppressed by thiamine but not iron (Figure 1B). Similarly, a *NEO1/neo1Δ thi3Δ/thi3Δ* mutant was no more sensitive than the *thi3Δ/thi3Δ* mutant to CQ (Figure S4). These results together suggested that CQ likely inhibits at least three independent biological processes in yeast: vacuolar functions, iron homeostasis, and thiamine metabolism or thiamine-dependent functions.

### Genome-wide GGSL and DS screens identified Thi7 as a candidate target of CQ

To further elucidate the MOA(s) of CQ that underlie the hypersensitivity of the *thi3Δ* mutant, we performed a genome-wide GGSL screen. We reasoned that a *thi3Δ* mutant is hypersensitive to CQ because Thi3 is required to functionally compensate for inactivation of the drug target. A genome-wide GGSL screen with *thi3Δ*, which discovers functional compensation between genes in an unbiased manner, could reveal such a target or components of a target pathway. Among 5 genes discovered (Table S2), deleting the high affinity thiamine transporter Thi7 [12] caused severe growth defects, although not lethality, in the *thi3Δ* mutant (Figure 1C), possibly as a consequence of reduction in both thiamine synthesis and uptake in the double mutant. This double mutant was still viable, likely due to the expression of two low-affinity thiamine transporters Nrt1 and Thi72 [13]. Although a *thi7Δ* mutant was no more sensitive to CQ than a wild-type strain, a *thi3Δ thi7Δ* double mutant was much more sensitive than the *thi3Δ* single mutant (Figure 1D). In addition, thiamine suppressed CQ-hypersensitivity of this *thi3Δ thi7Δ* mutant, and the amount of thiamine needed for such suppression roughly correlated with the amount of CQ in the media (Figure 1E). Significantly, the CQ concentration (i.e., 20 µM) needed to completely inhibit growth of the *thi3Δ thi7Δ* double mutant was also achievable in human patients, animal studies or human cell culture experiments, suggesting that the underlying MOA, if conserved, are likely medically relevant.

In parallel, we performed a dosage suppression (DS) screen for genes that would suppress the CQ-hypersensitivity of the *thi3Δ* mutant. Such a screen could also discover a drug target or component of a target pathway. In order to increase specificity, we performed the screen first in the *thi3Δ thi7Δ* double mutant, which is much more sensitive to CQ than the *thi3Δ* single mutant, and subsequently tested candidate suppressors in the single mutant. We thought that screening in the double mutant would permit the use of a relatively low dose of CQ (i.e., 20 µM) and potentially minimize inhibition of other pathways. The screen identified *THI3*, *THI7*, *THI20*, and *PDC2* (Figure 1F and data not shown). We subsequently showed that overexpression of *THI7* and *PDC2* also suppressed CQ-hypersensitivity of the *thi3Δ* single mutant at a higher CQ concentration (Figure 1G). However, the effect of *PDC2* overexpression under this condition largely depended on *THI7* (Figure 1F and 1G), consistent with a previous report that *PDC2* controls expression of *THI7* and thiamine biosynthesis genes [13]. Taken together, both the GGSL and DS screens in the *thi3Δ* mutant background discovered Thi7, suggesting that it might be a target of CQ.

### CQ likely inhibits both high- and low-affinity thiamine transporters in yeast

**Figure 2:**
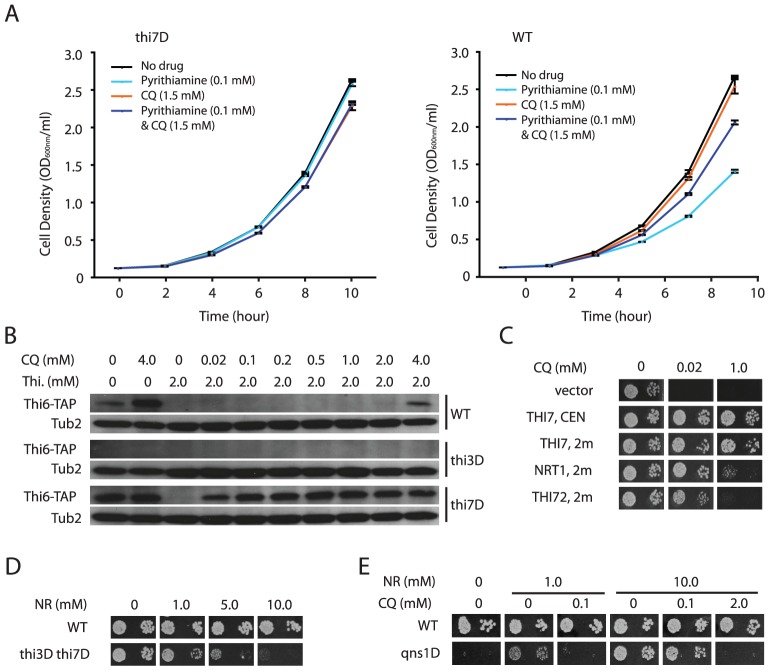
CQ-treatment mimics the effects of inactivating thiamine transporters. A. CQ partially suppresses Thi7-dependent toxicity of pyrithiamine. Growth curves of a wild-type (WT) strain and a *thi7Δ* mutant under the indicated conditions were plotted. B. Thi3-dependent induction of expression of a Thi6-TAP fusion protein by CQ-treatment, a *thi7Δ* mutation, and thiamine deprivation. The fusion protein was detected with an anti-TAP antibody. Tub2 was used as a loading control. C. Suppression of CQ-hypersensitivity of the *thi3Δ thi7Δ* double mutant by expressing *THI3*, *THI7*, *NRT1*, or *THI72* from a centromeric (*CEN*) or high copy (*2µ*) plasmid. D. Excess amount of nicotinamide riboside (NR) inhibits growth of the *thi3Δ thi7Δ* double mutant. E. CQ inhibits NR-dependent growth of a *qns1Δ* mutant.

To investigate such a possibility, we tested whether CQ affects other phenotypes controlled by Thi7. Thi7 was previously shown to be required for the uptake and toxicity of pyrithiamine in yeast [12], and as expected, a *thi7Δ* mutant was resistant to pyrithiamine (Figure 2A). Consistent with our model, CQ also partly suppressed the toxic effect of pyrithiamine in a wild-type strain (Figure 2A). Furthermore, thiamine deprivation due to *thi7Δ* mutation or the lack of thiamine in growth medium was previously shown to induce expression of thiamine biosynthesis genes in a Thi3-dependent manner [13]. We found that CQ treatment induces Thi3-dependent expression of thiamine biosynthesis genes *THI6* and *THI11* (Figure 2B and data not shown), indicating that it causes thiamine deficiency. These results were consistent with the model that CQ inhibits Thi7.

However, the *thi7Δ* mutation further enhanced the effect of CQ treatment on *THI6* expression (Figure 2B), suggesting that CQ likely also inhibit additional targets to augment thiamine deficiency. In addition to Thi7, the yeast genome encodes two low affinity thiamine transporters Nrt1 and Thi72, which share high sequence identity (>84%) with Thi7 [13]. Possibly, CQ also inhibits these two thiamine transporters, a model consistent with the observation that the *thi3Δ thi7Δ* double mutant is viable but much more sensitive to CQ than the *thi3Δ* single mutant (Figure 1D). Presumably, it takes much less CQ to inhibit thiamine uptake through these low affinity transporters. This model was further supported by the observation that both Nrt1 and Thi72 confer CQ-resistance in the *thi3Δ thi7Δ* double mutant when overexpressed from a high copy plasmid under control of the *THI7* promoter (Figure 2C). To further corroborate this model, we took advantage of the fact that Nrt1 is also a high affinity transporter for nicotinamide riboside (NR) [21], and tested if growth of the *thi3Δ thi7Δ* mutant is also inhibited by NR. Similar to CQ, 10 µM of NR impaired growth of the *thi3Δ thi7Δ* double mutant in the presence of 1 µM of thiamine (Figure 2D), indicating that Nrt1 is at least partly responsible for thiamine uptake in this strain. We next tested whether CQ impairs the function of Nrt1. By taking advantage of the observation that a *qns1Δ* mutant requires Nrt1-dependent uptake of exogenously supplied NR for survival [21], [22], we found that CQ inhibited NR-dependent growth of such a *qns1Δ* mutant, and that the amount of CQ needed for growth inhibition roughly correlated with the amount of NR present in the medium (Figure 2E). These results together strongly suggested that CQ inhibits both the high- and low-affinity thiamine transporters in yeast.

### CQ inhibits thiamine uptake via wild-type Thi7

**Figure 3:**
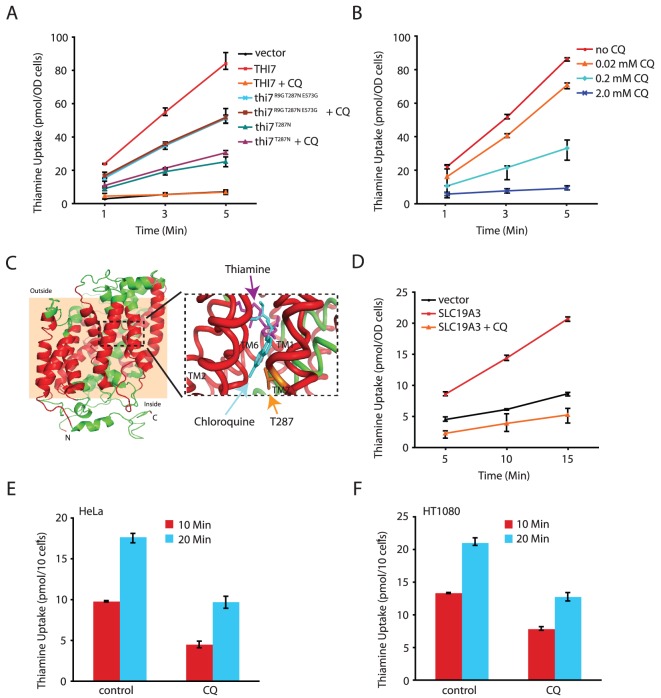
CQ inhibits thiamine transporters from yeast and human. A. CQ (1.0 mM) completely inhibits thiamine uptake mediated by wild-type Thi7 but not CQ-resistant mutants of indicated genotypes. B. CQ dose-dependent inhibition of the transporter activity of wild-type Thi7. C. A homology model of the closed form of Thi7. The transmembrane (TM) helices are shown in red with the membrane region shown in a peach background. The black dashed rectangle is shown on the right in detail with docked substrates. The lowest energy docking results of thiamine (magenta) and chloroquine (cyan) are shown, with the side chain of T287 depicted in orange. D. CQ (1.0 mM) completely inhibits the human thiamine transporter SLC19A3 expressed in yeast cells. E and F. CQ (0.25 mM) inhibits thiamine uptake in human HeLa (E) and HT1080 (F) cell lines.

We next directly tested the model that CQ inhibits thiamine transporters using a well-defined uptake assay [23]. As expected, thiamine uptake in the *thi3Δ thi7Δ* mutant was undetectable, but this was restored with expression of wild-type *THI7* from a plasmid (Figure 3A). CQ blocked thiamine uptake mediated by the wild-type Thi7 transporter in a dose-dependent manner (Figure 3A and 3B). In contrast, it completely failed to inhibit thiamine uptake mediated by a CQ-resistant Thi7 allele (Thi7^R9G T287N E573G^) (Figure 3A) isolated from screening a *THI7* random mutagenesis library expressed in the *thi3Δ thi7Δ* double mutant. This allele conferred higher levels of CQ-resistance as compared to wild-type *THI7* when expressed in the *thi3Δ thi7Δ* double mutant (Figure S5). We subsequently found that the T287N substitution was largely responsible for the resistance phenotype of this mutant (Figure 3A and Figure S5). These results together indicated that CQ directly inhibits the thiamine transporter activity of yeast Thi7.

To gain further insights into how CQ might inhibit thiamine uptake through Thi7, we performed 3D- homology modeling of Thi7 using the crystal structure of the substrate bound benzyl-hydantoin transporter Mhp1 from *Microbacterium liquefaciens* [24] as a template. A model of correct topology and close structural homology was obtained as judged by confidence (C) and template modeling (TM) scores of 0.87 and 0.83, respectively. Analogous to the Mhp1 structure [24], the transmembrane (TM) helices 1, 2, 6, and 7 of the Thi7 model form a four-helix bundle that harbors a putative substrate-binding site. The CQ-resistance T287N mutation was mapped to TM7 (Figure 3C). Importantly, both thiamine and CQ could be docked into the substrate-binding site of Thi7 with high affinity (Figure 3C and Table S3). We thus tentatively conclude that CQ might compete with thiamine for binding to the transporter. Such a model is also consistent with the observation that excess amount of thiamine suppresses the inhibitory effect of CQ on cellular growth (Figure 1B and 1E). Based on the docking results, the CQ-resistant T287N mutation is located closer to CQ than to thiamine (Figure 3C). However, at a distance of about 6.1 Å, a close interaction between this residue and CQ does not seem possible. In addition, mutating this Thr287 residue to Ala, Asp, Gln, and Ile did not confer CQ-resistance (data not shown). It is possible that the T287N mutation affects the conformation of the substrate-binding site of the transporter, a hypothesis also consistent with the observation of partial reduction in the thiamine uptake activity of the mutant even in the absence of CQ (Figure 3A). However, understanding how the T287N mutation completely abolishes the inhibitory effect of CQ on thiamine uptake will likely require a crystal structure of Thi7.

### CQ inhibits a human thiamine transporter

We next investigated whether CQ inhibition of thiamine transporters is conserved. Human cells express two thiamine transporters SLC19A2 and SLC19A3 that are ∼70% identical in amino acid sequences [14], [15]. We found that expression of human SLC19A3, which share ∼15% sequence identity with Thi7, partly restored thiamine uptake in the *thi3Δ thi7Δ* double mutant (Figure 3D). Importantly, CQ almost completely inactivated such an activity (Figure 3D). Moreover, CQ significantly inhibited thiamine uptake in two human cell lines tested (Figure 3E and 3F). These results together demonstrated that inhibition of thiamine transporters and reduction in thiamine uptake is a conserved MOA of CQ in both yeast and human.

### Other quinoline-containing antimalarials also inhibit thiamine transporters

**Figure 4:**
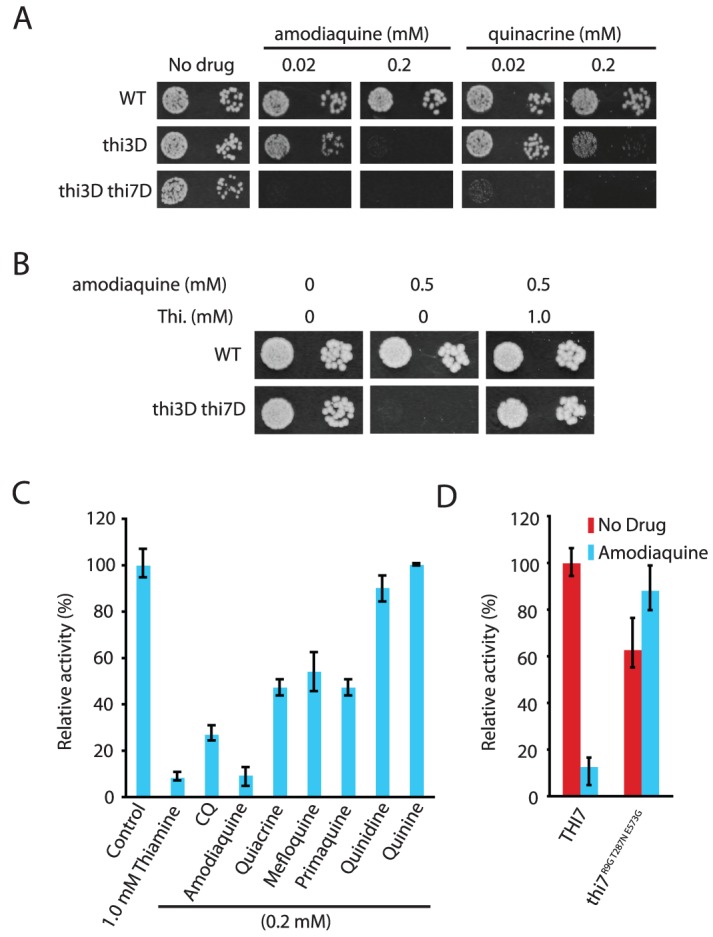
Other antimalarials inhibit thiamine transporters. A. Hypersensitivity of the *thi3Δ* and *thi3Δ thi7Δ* mutants to amodiaquine and quinacrine. B. Suppression of amodiaquine-hypersensitivity of the *thi3Δ* and *thi3Δ thi7Δ* mutants by exogenously supplied thiamine. C. Inhibition of thiamine uptake by indicated compounds. D. Amodiaquine (0.25 mM) inhibits thiamine uptake mediated by wild-type Thi7 but not a CQ-resistant Thi7^R9G T287N E573G^ mutant.

We next asked whether other quinoline-containing antimalarials also inhibit thiamine uptake through the transporters. When applied at 0.2 mM, amodiaquine, quinacrine, mefloquine, primaquine, quinine, and quinidine all inhibited growth of a *thi3Δ thi7Δ* mutant (Figure 4A and Figure S6). Some of them also inhibited growth of the *thi3Δ* single mutant (Figure 4A). Similar to CQ, the inhibitory effects of these other antimalarials on cellular growth were suppressed by excess amount of thiamine in the medium (Figure 4B and data not shown). Most of these other antimalarials also inhibited thiamine uptake mediated by the wild-type Thi7, with amodiaquine having the strongest effect (Figure 4C). Similar to CQ, amodiaquine completely failed to inhibit thiamine uptake mediated by the Thi7^R9G T287N E573G^ mutant (Figure 4D). These results together suggested that inhibition of thiamine uptake is a conserved mechanism among quinoline-containing antimalarials.

## Discussion

In this study, we demonstrated that CQ and other quinoline-containing antimalarials inhibit thiamine transporters in yeast. We also showed that such a MOA is conserved between yeast and humans. In particular, the human thiamine transporter SLC19A3 was completely inhibited by CQ when expressed in yeast cells (Figure 3D). This MOA is likely medically relevant. First, much like the *thi3Δ* yeast mutant, human cells completely depend on exogenously supplied thiamine for survival, and the thiamine transporters play essential roles in this process. Second, at 20 µM, a concentration achievable in human patients, CQ completely inhibited growth of the *thi3Δ thi7Δ* double mutant (Figure 2B). The concentration need to significantly inhibit the *thi3Δ* single mutant was about 10 times higher, but yeast cells are generally known to be more resistant to many drugs than mammalian cells due to the presence of cell wall and potent drug pumps. Third, the concentration of thiamine in human serum is in the 10–20 nM range [25], [26], [27], more than two-magnitude lower than those used in this study. The putatively competitive relationship between CQ and thiamine suggests that inhibition of thiamine uptake in human body is achievable using CQ concentrations much lower than those used in this study. Fourth, CQ accumulates in certain tissues (e.g. the retina) at high concentrations, an observation particularly relevant to retinopathy caused by CQ-based medications [4], [28], [29]. In this regard, there is already a connection between thiamine deficiency and retinopathy in diabetic patients [30], and diabetic retinopathy can be prevented with thiamine supplementation in a rodent animal model [31]. In addition, thiamine deficiency and CQ treatment both lead to neurological and cardiovascular disorders [5], [6], [32], [33]. Based on these, it will be interesting to investigate whether thiamine deficiency might underlie some of the CQ-induced adverse effects and whether these can be prevented with concomitant thiamine supplementation.

**Figure 5:**
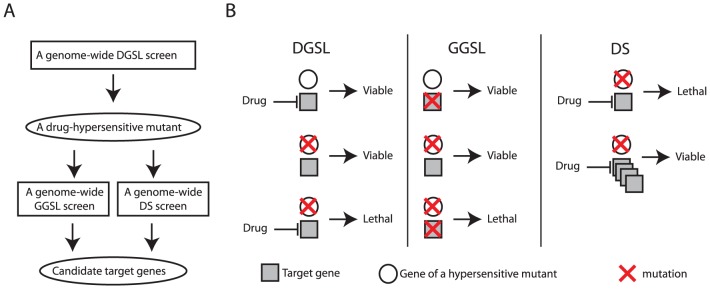
A diagram and concept of discovering drug target using the SL/DS strategy. A flow chart (A) and concept (B) of drug target identification with a combination of DGSL, GGSL, and DS screens.

This study also demonstrated SL/DS as a novel and effective functional genomics strategy for discovering drug targets. This strategy starts with identifying mutants that are hypersensitive specifically to a drug treatment with a genome-wide DGSL screen (Figure 5A). Such a drug-hypersensitive mutant (e.g, *thi3Δ*) is then used as a key to directly discover drug target(s) with a genome-wide GGSL or DS screen, or both (Figure 5A). Discovering a drug target with a subsequent GGSL screen is based on the premise that genetic and pharmacological inactivation of a drug target produce similar effects (e.g., fitness defect in the hypersensitive mutant) (Figure 5B). Discovering a drug target with a subsequent DS screen is based on the principle that overexpressing a drug target confers drug resistance [34], in this case, in a hypersensitive mutant (Figure 5B). That both GGSL and DS screens identified Thi7 greatly simplified its selection as a high likelihood candidate CQ target for validation. We note that the particular DS screen reported in this study was performed in the *thi3Δ thi7Δ* double mutant, with an intention of using a low dose of CQ to potentially minimize inhibition of additional targets to increase pathway specificity. Such a DS screen would probably have also succeeded if a *thi3Δ* single mutant had been used.

Most existing *in vivo* target identification methods such as haploinsufficiency profiling [16], outright dosage suppression [35], and discovering resistance mutations with genome-sequencing or high throughput complementation [36], [37] typically rely on a drug's ability to completely or severely inhibit growth of wild-type cells. In contrast, SL/DS does not have such a requirement and thus can be used to discover non-essential genes as drug targets, as shown with Thi7 in this study. This feature is very significant considering that >80% of all proteins encoded by the yeast genome are non-essential. As a result, this method will offer much broader opportunity than the existing methods for discovering drug targets, especially with drugs that inhibit the growth of certain mutants but not wild-type cells.

SL/DS should also be useful in discovering essential proteins as drug targets. In this regard, it may not be as straightforward as the other methods. However, we have found that the existing methods fail to discover targets of many cytotoxic drugs (unpublished). A possible reason for that is that some drugs simultaneously inhibit multiple targets and that, consequentially, overexpressing or mutating any single target gene does not confer drug resistance in an otherwise wild-type strain background. In such a case, SL/DS could be effective because it is always possible to first discover drug-hypersensitive deletion mutants using a DGSL screen and subsequently identify the drug targets using GGSL and DS screens in these mutant backgrounds. The DS screen in a hypersensitive mutant could work because a lower drug dose can be used to minimize inhibition of other target pathways.

The SL/DS methodology seems to be similar to but is distinctly different from a previously described compendium approach, where targets of novel drugs are inferred from comparing a large compendium of genome-wide DGSL profiles of old drug treatments and GGSL profiles of genetic perturbation for similarities [38]. Like SL/DS, this compendium approach could identify both essential and non-essential proteins as drug targets using the DGSL and GGSL profiles [38]. However, it does not directly identify drug targets but instead infers candidate targets from profile similarity. A potential limitation is that perturbations in potentially many components of a given drug target pathway typically produce similar profiles, making it difficult to determine the actual drug target. Its discovery scope is also limited to the available DGSL or GGSL reference profiles, which are very difficult to generate at a large scale in higher eukaryotes. In contrast, SL/DS directly identifies a drug's target with only three genome-wide screens: DGSL followed with GGSL and DS. It does not rely on DGSL profiles of other drugs or GGSL profiles of other genetic perturbation as references. A similar SL/DS strategy will likely also be useful for drug target identification in human cells, where genome-wide DGSL, GGSL, and DS screens are now possible [39], [40], [41], [42].

## Methods

### Strains and plasmids

Yeast strains used in this study include the wild-type strains BY4741**a** (*MAT* **a** *his3Δ1 leu2Δ0 lys2Δ0 ura3Δ0*) and BY4743**a**/α (*MAT* **a**/α *his3Δ1/his3Δ1 leu2Δ0/leu2Δ0 LYS2/lys2Δ0 met15Δ0/MET15 ura3Δ0/ura3Δ0*) [43] and isogenic mutants derived from a genome-wide deletion library [44]. The haploid-convertible heterozygous diploid deletion library used in screening for CQ hypersensitive mutants was previously described [44]. The Thi6-TAP and Thi11-TAP strains were obtained from Open Biosystems and the derivative *thi7Δ::kanMX* and *thi3Δ::natMX* mutants were constructed by deleting the *THI7* and *THI3* genes, respectively in these strain backgrounds. Bacterial strain DH5α was used as the host during molecular cloning. Plasmids used in this study are listed in Table S4. The vectors used are pRS416 [43] and YEplac195 [45].

### Yeast media and chemicals

Yeast media used in this study include a haploid selection synthetic complete medium SC−Leu−His−Arg+G418+canavanine [46] and a synthetic complete (SC) medium that either contained or lacked uracil (SC-Ura) or leucine (SC-Leu). Amodiaquine dihydrochloride, Chloroquine diphosphate, mefloquine hydrochloride, primaquine bisphosphate, pyrithiamine hydrobromide, quinacrine dihydrochloride, quinine, quinidine, thiamine hydrochloride, and ferric chloride were all purchased from Sigma. Nicotinamide riboside (NR) was freshly derived from enzymatic hydrolysis of NMN (Sigma) and quantified as previously described [22]. Stock solutions were made in ddH_2_O with the help of adjusting pH when necessary, filter sterilized, and stored at −20°C or directly used.

### Genome-wide DGSL, GGSL, and DS screens

Genome-wide DGSL screens and subsequent individual validation were carried out as previously described [46], [47]. 5 mM of CQ was used in the screen. Concentrations of 0.5 mM, 1 mM, 2 mM, 3 mM, 4 mM, and 5 mM were used in validation assays to further distinguish among the sensitivities of different mutants. Genome-wide GGSL screen with a *thi3Δ::URA3* query construct was carried out as previously described [48], [49]. Candidate hits were validated with tetrad dissection. Briefly, the heterozygous diploid deletion mutant of each candidate gene was transformed with a *thi3Δ::URA3* query construct to disrupt one copy of *THI3*. The resultant heterozygous diploid double mutant was sporulated and the spores were dissected under a microscope on a YPD plate. The plate was incubated at 30°C for 2 days and photographed and the genotype of the dissected spores were determined by their growth on SC-Ura and YPD+G418 plates. Genome-wide DS screen in the *thi3Δ thi7Δ* double mutant was performed using a genome-wide tiling library containing ∼95% of the yeast genomic sequences [50]. This library was *en masse* transformed into XPY1263**a** (*MAT* **a** *thi3Δ::natMX thi7Δ::kanMX*). An aliquot of ∼10^5^ cells of the transformed pool was subsequently plated on a solid synthetic complete medium that lacked leucine (SC-Leu) but contained 20 µM CQ to select for resistant colonies. Plasmids were recovered from 96 representative colonies and sequenced at one end to identify the responsible genes. Candidate genes were then individually validated on YEplac195 for their ability to confer CQ-resistance in the *thi3Δ thi7Δ* double and *thi3Δ* single mutants.

### Western blotting

A yeast strain expressing Thi6-TAP or Thi11-TAP from the endogenous locus in a wild-type, *thi3Δ*, or *thi7Δ* background was grown in 5 ml of regular liquid SC at 30°C for an overnight. Cells were harvested, washed with 5 ml of sterile water, and inoculated into 5 ml of liquid SC that lacked thiamine at an starting cell density of ∼0.15 OD_600 nm_/ml. CQ and thiamine were added at the indicated final concentrations. The cultures were incubated at 30°C for 4 hr with shaking. About 1.0 OD_600 nm_ cells were collected for each sample, directly lysed with boiling in 1× SDS buffer, and analyzed with western blot using an anti-TAP antibody (Open biosystems) and an anti-Tub2 antibody.

### Thiamine uptake assays

Thiamine uptake in yeast cells was carried out as described [23] with minor modifications. Yeast cells of XPY1263**a** harboring an empty vector or expressing wild-type *THI7*, *thi7^R9G T287N E573G^*, *thi7^T287N^*, or SLC19A3 were grown in 3 ml liquid SC-Ura containing 100 uM at 30°C for overnight. 1 ml of each overnight culture was inoculated into 50 ml of fresh SC-Ura liquid and incubated at 30°C for 4.5 hrs. Cells were harvested, washed twice each with 10 ml of ddH_2_O, and suspended in citric acid/phosphate buffer (pH 4.5) containing 1% D-glucose at a density of 2.0–2.5 OD_600_ nm/ml. For each uptake experiment, 500 µl of cells were pre-warmed at 30°C for 3 min in a microcentrifuge tube in the presence or absence of CQ or another antimalarial at indicated concentrations. [H^3^]-Thiamine (American Radiochemical Company) was added at a final concentration of 2 µM and a specificity of 0.2 Ci/mmol and immediately mixed on a Mixmate at 30°C. 100 µl of each sample was taken at indicated time points (1 min, 3 min, and 5 min) and transferred to a microcentrifuge tube that contains 900 µl of ice-cold 1 mM thiamine in citric acid/phosphate buffer (pH 4.5) to terminate uptake of H^3^]-Thiamine. Cells were collected by filtering and washed with 10 ml of ddH_2_O. Radioactivity associated with each filter was measured with a Beckman scintillation counter and used to calculate thiamine uptake activity as pmol/OD_600 nm_ cells. Three independent repeats were performed for each time point and the results were averaged.

Thiamine uptake in human cells was carried out as described in another previous study [51] with minor modifications. HeLa and HT1080 cells were grown in DMEM medium until confluent monolayers in 12-well plates, with ∼5.0×10^5^ cells in each well. Medium was aspirated 4 days following confluence, and each culture was washed twice with the uptake buffer (NaCl, 125 mM; KCl, 4.8 mM; KH_2_PO_4_, 1.2 mM; MgSO_4_, 1.2 mM; CaCl_2_, 1.2 mM; Glucose, 5 mM; Glutamine, 5 mM; HEPES-NaOH, 12.5 mM; MES, 12.5 mM; pH 8.0) that had been pre-warmed at 37°C. Cell monolayers were then pre-incubated in 0.2 ml uptake buffer that either contained or lacked CQ at 0.25 mM at 37°C for 10 min. H^3^]-Thiamine was subsequently added at a final concentration of 5 µM and a specificity of 1 Ci/mmol. [H^3^]-Thiamine uptake was terminated at 10- or 20-min time point by addition of 1 ml of ice-cold buffer that contained 1 mM of unlabeled thiamine into each well. Buffer was immediately aspirated. Cells from each well were rinsed twice with 1 ml of ice-cold buffer containing unlabeled thiamine, digested with 0.25 ml of 1 N NaOH for 2 hours, and neutralized with 0.25 ml of 1 N HCl. Cell lysates (∼0.5 ml each) were transferred into scintillation vials. Residual lysate in each well was washed with 0.3 ml of stoppage buffer and also transferred to the same scintillation vials. Radioactivity of each sample was measured with a Beckman scintillation counter and used to calculate thiamine uptake activity as pmol/10^6^ cells. Three independent repeats were performed for each time point and the results were averaged.

### Homology modeling of Thi7

Coordinates of the substrate bound form of the benzyl-hydantoin transporter Mhp1 structure from *Microbacterium liquefaciens*, (PDB code :2JLO) [24]. was used as a starting template to obtain a structural model of the yeast Thi7 through the online server I-TASSER (http://zhanglab.ccmb.med.umich.edu/I-TASSER/) [52]. Thi7 has 21% sequence identity and 35% similarity with the benzyl-hydantoin transporter Mhp1 from *Microbacterium liquefaciens*. The Thi7 residues from A22 to E537 were used as the input sequence based on a BLAST sequence analysis with the Mhp1 sequence. Sequence template alignments were generated using the program MUSTER, which is built into I-TASSER. The quality of the generated model was assessed in I-TASSER based on two major criteria, the C- and the TM-scores.

### Docking of thiamine and CQ into the Thi7 model

Thiamine (Pubchem ID: 1130) and chloroquine (Pubchem ID: 2719) were processed for docking using ADT tools. Addition of hydrogen atoms and setting of rotatable bonds for these substrates were carried out in ADT tools (Molecular graphics lab of the Scripps research institute). The docking of substrates to the Thi7 model was performed using the AutoDockVina software [53]. A grid box with a dimension of 15×15×15 points was used.

## Supporting Information

Figure S1 CQ-hypersensitive heterozygous diploid deletion mutants identified from a genome-wide screen. A. Growth of mutants of indicated genotypes in the presence or absence of CQ of indicated concentrations. B. Complementation of CQ-hypersensitivity of a *NEO1/neo1Δ* mutant with expressing *NEO1* from a high copy plasmid.
(EPS)

Figure S2 GO terms enriched for CQ-hypersensitive haploid deletion mutants. Gene lists identified from the genome-wide DGSL screen were subject to DAVID for functional analysis. Significantly enriched cellular functions were selected based on a cutoff Q value of 0.05 and plotted.
(EPS)

Figure S3 A non-additive relationship between the *fet3Δ* and *thi3Δ* mutations for their effects on CQ-sensitivity. Haploid strains of indicated genotypes were grown in the presence or absence of CQ (0.5 mM).
(EPS)

Figure S4 Non-additive effects of the *NEO1/neo1Δ* and *thi3Δ/thi3Δ* mutations on CQ-hypersensitivity. Isogenic diploid strains of indicated genotypes were grown in the presence of absence of CQ at indicated concentrations.
(EPS)

Figure S5 A T287N mutation on Thi7 confers CQ-resistance. Growth of a *thi3Δ thi7Δ* double mutant expressing centromeric plasmids of indicated genotypes in the presence or absence of CQ at indicated concentrations.
(EPS)

Figure S6 Other quinoline-containing antimalarials inhibit growth of a thi3*Δ* thi7*Δ* double mutant. Isogenic haploid strains of indicated genotype were grown in the presence or absence of 0.2 mM of indicated drugs.
(EPS)

Table S1 Chloroquine hypersensitive haploid deletion mutants. Haploid-convertible heterozygous diploid eletion mutants of the listed genes were sporulated on a solid sporulation medium and spotted as 10× serial dilution on a haploid selection medium that either contained or lacked CQ of indicated concentration. Growth of each haploid deletion mutant was inspected and scored, with its growth in the absence of the drug as a reference. S–sick; L–Lethal.
(DOC)

Table S2 Genes identified from a genome-wide *thi3Δ* synthetic lethality screen. Heterozygous diploid double mutants of between the listed genes and *THI3* were constructed and subjected to tetrad analysis. Synthetic sick interactions were observed in all cases.
(DOC)

Table S3 Docking results of thiamine or chloroquine into a 3D-model of Thi7. Both thiamine and chloroquine were docked into a 3D-model of Thi7 using ADT tools. Results of top 9 docking modes were listed.
(DOC)

Table S4 Plasmids used in this study.
(DOC)
