# Supplementary material for: Discovering Thiamine Transporters as Targets of Chloroquine Using a Novel Functional Genomics Strategy
Source: PLoS Genet. 2012 Nov 29;8(11):e1003083. doi: 10.1371/journal.pgen.1003083 (PMC3510038; doi:10.1371/journal.pgen.1003083)
Supplement: Table S1 — Chloroquine hypersensitive haploid deletion mutants. Haploid-convertible heterozygous diploid eletion mutants of the listed genes were sporulated on a solid sporulation medium and spotted as 10× serial dilution on a haploid selection medium that either contained or lacked CQ of indicated concentration. Growth of each haploid deletion mutant was inspected and scored, with its growth in the absence of the drug as a reference. S–sick; L–Lethal. (DOC) [file pgen.1003083.s007.doc]

| **Table S1. Chloroquine hypersensitive haploid deletion mutants.** | | | | | | |
| --- | --- | --- | --- | --- | --- | --- |
| **ORF** | **Gene** | **Chloroquine concentration** | | | | |
|  |  | **1 mM** | **2 mM** | **3 mM** | **4 mM** | **5 mM** |
| *YMR058W* | *FET3* | L | L | L | L | L |
| *YER145C* | *FTR1* | L | L | L | L | L |
| *YKL184W* | *SPE1* | L | L | L | L | L |
| *YDL080C* | *THI3* | L | L | L | L | L |
| *YCR094W* | *CDC50* | S | L | L | L | L |
| *YDL185W* | *TFP1* | S | L | L | L | L |
| *YEL027W* | *CUP5* | S | L | L | L | L |
| *YEL051W* | *VMA8* | S | L | L | L | L |
| *YHR026W* | *PPA1* | S | L | L | L | L |
| *YJL204C* | *RCY1* | S | L | L | L | L |
| *YLR447C* | *VMA6* | S | L | L | L | L |
| *YNL280C* | *ERG24* | S | L | L | L | L |
| *YNL296W* |  | S | L | L | L | L |
| *YNL297C* | *MON2* | S | L | L | L | L |
| *YOR332W* | *VMA4* | S | L | L | L | L |
| *YPL234C* | *TFP3* | S | L | L | L | L |
| *YJL176C* | *SWI3* | S | S | S | S | L |
| *YBR127C* | *VMA2* |  | L | L | L | L |
| *YCL007C* | *CWH36* |  | L | L | L | L |
| *YGR020C* | *VMA7* |  | L | L | L | L |
| *YKL080W* | *VMA5* |  | L | L | L | L |
| *YKL119C* | *VPH2* |  | L | L | L | L |
| *YAL026C* | *DRS2* |  | S | L | L | L |
| *YGR105W* | *VMA21* |  | S | L | L | L |
| *YHR060W* | *VMA22* |  | S | L | L | L |
| *YKL118W* |  |  | S | L | L | L |
| *YMR202W* | *ERG2* |  | S | L | L | L |
| *YPR123C* |  |  | S | L | L | L |
| *YPR124W* | *CTR1* |  | S | L | L | L |
| *YDR123C* | *INO2* |  | S | S | S | L |
| *YJL117W* | *PHO86* |  | S | S | L | L |
| *YLR242C* | *ARV1* |  | S | S | S | S |
| *YLR396C* | *VPS33* |  | S | S | L | L |
| *YMR231W* | *PEP5* |  | S | S | L | L |
| *YOL108C* | *INO4* |  | S | S | S | S |
| *YDR269C* |  |  |  | L | L | L |
| *YML008C* | *ERG6* |  |  | L | L | L |
| *YNL243W* | *SLA2* |  |  | L | L | L |
| *YBR289W* | *SNF5* |  |  | S | L | L |
| *YDR270W* | *CCC2* |  |  | S | L | L |
| *YDR455C* |  |  |  | S | L | L |
| *YDR456W* | *NHX1* |  |  | S | L | L |
| *YHR030C* | *SLT2* |  |  | S | L | L |
| *YKL212W* | *SAC1* |  |  | S | L | L |
| *YPL045W* | *VPS16* |  |  | S | L | L |
| *YBL071C* |  |  |  | S | S | S |
| *YGL007W* |  |  |  | S | S | S |
| *YHL025W* | *SNF6* |  |  | S | S | S |
| *YJL029C* | *VPS53* |  |  | S | S | L |
| *YJL056C* | *ZAP1* |  |  | S | S | S |
| *YJL175W* |  |  |  | S | S | S |
| *YKL139W* | *CTK1* |  |  | S | S | S |
| *YLR191W* | *PEX13* |  |  | S | S | S |
| *YMR123W* | *PKR1* |  |  | S | S | S |
| *YPR163C* | *TIF3* |  |  | S | S | S |
| *YAL012W* | *CYS3* |  |  |  | L | L |
| *YCR044C* | *PER1* |  |  |  | L | L |
| *YNL238W* | *KEX2* |  |  |  | L | L |
| *YBR036C* | *CSG2* |  |  |  | S | L |
| *YDR484W* | *SAC2* |  |  |  | S | L |
| *YGR133W* | *PEX4* |  |  |  | S | L |
| *YIL154C* | *IMP2'* |  |  |  | S | L |
| *YJL095W* | *BCK1* |  |  |  | S | L |
| *YJR040W* | *GEF1* |  |  |  | S | L |
| *YJR073C* | *OPI3* |  |  |  | S | L |
| *YLR025W* | *SNF7* |  |  |  | S | L |
| *YNR023W* | *SNF12* |  |  |  | S | L |
| *YPL214C* | *THI6* |  |  |  | S | L |
| *YDR027C* | *LUV1* |  |  |  | S | S |
| *YGL167C* | *PMR1* |  |  |  | S | S |
| *YGL168W* | *HUR1* |  |  |  | S | S |
| *YLR056W* | *ERG3* |  |  |  | S | S |
| *YLR087C* | *CSS1* |  |  |  | S | S |
| *YLR240W* | *VPS34* |  |  |  | S | S |
| *YLR417W* | *VPS36* |  |  |  | S | S |
| *YMR165C* | *SMP2* |  |  |  | S | S |
| *YMR242C* | *RPL20A* |  |  |  | S | S |
| *YNL322C* | *KRE1* |  |  |  | S | S |
| *YNL329C* | *PEX6* |  |  |  | S | S |
| *YOR198C* | *BFR1* |  |  |  | S | S |
| *YPL031C* | *PHO85* |  |  |  | S | S |
| *YBL058W* | *SHP1* |  |  |  |  | S |
| *YBR240C* | *THI2* |  |  |  |  | S |
| *YCL008C* | *STP22* |  |  |  |  | S |
| *YFL025C* | *BST1* |  |  |  |  | S |
| *YGR166W* | *KRE11* |  |  |  |  | S |
| *YHR064C* | *SSZ1* |  |  |  |  | S |
| *YJR102C* | *VPS25* |  |  |  |  | S |
| *YKR001C* | *VPS1* |  |  |  |  | S |
| *YML112W* | *CTK3* |  |  |  |  | S |
| *YMR077C* | *VPS20* |  |  |  |  | S |
| *YMR238W* | *DFG5* |  |  |  |  | S |
| *YOR030W* | *DFG16* |  |  |  |  | S |
| *YOR039W* | *CKB2* |  |  |  |  | S |
| *YPL002C* | *SNF8* |  |  |  |  | S |
