# Supplementary material for: Discovering Thiamine Transporters as Targets of Chloroquine Using a Novel Functional Genomics Strategy
Source: PLoS Genet. 2012 Nov 29;8(11):e1003083. doi: 10.1371/journal.pgen.1003083 (PMC3510038; doi:10.1371/journal.pgen.1003083)
Supplement: Table S2 — Genes identified from a genome-wide thi3Δ synthetic lethality screen. Heterozygous diploid double mutants of between the listed genes and THI3 were constructed and subjected to tetrad analysis. Synthetic sick interactions were observed in all cases. (DOC) [file pgen.1003083.s008.doc]

| **Table S2. Genes identified from a genome-wide *thi3∆* synthetic lethality screen.** | |
| --- | --- |
| **Gene** | **Molecular Function** |
| *DAP1* | Heme-binding protein involved in regulation of ergosterol biosynthesis |
| *SAP30* | Subunit of a histone deacetylase complex involved in gene silencing |
| *SDS3* | Subunit of a histone deacetylase complex involved in gene silencing |
| *RXT2* | Subunit of a histone deacetylase complex involved in gene silencing |
| *THI7* | High affinity transporter responsible for thiamine uptake |
