# Supplementary material for: Discovering Thiamine Transporters as Targets of Chloroquine Using a Novel Functional Genomics Strategy
Source: PLoS Genet. 2012 Nov 29;8(11):e1003083. doi: 10.1371/journal.pgen.1003083 (PMC3510038; doi:10.1371/journal.pgen.1003083)
Supplement: Table S3 — Docking results of thiamine or chloroquine into a 3D-model of Thi7. Both thiamine and chloroquine were docked into a 3D-model of Thi7 using ADT tools. Results of top 9 docking modes were listed. (DOC) [file pgen.1003083.s009.doc]

| **Table S3.** **Docking results of thiamine or chloroquine into a 3D-model of Thi7.** | | | | | | | |
| --- | --- | --- | --- | --- | --- | --- | --- |
| Thiamine | | | | Chloroquine | | | |
| Mode | Affinity  kcal/mol | Distance from rmsd | Best mode rmsd | Mode | Affinity  kcal/mol | Distance from rmsd | Best mode rmsd |
| 1 | -6.4 | 0 | 0 | 1 | -7.1 | 0 | 0 |
| 2 | -6.2 | 16.44 | 18.746 | 2 | -7.1 | 0.986 | 1.703 |
| 3 | -6.2 | 1.914 | 3.287 | 3 | -7.0 | 14.51 | 18.268 |
| 4 | -6.1 | 9.901 | 11.861 | 4 | -7.0 | 1.595 | 7.085 |
| 5 | -6.0 | 1.921 | 2.314 | 5 | -7.0 | 14.474 | 17.856 |
| 6 | -6.0 | 9.618 | 11.978 | 6 | -7.0 | 14.518 | 18.077 |
| 7 | -5.9 | 4.245 | 6.752 | 7 | -6.9 | 14.881 | 18.7 |
| 8 | -5.9 | 1.841 | 3.049 | 8 | -6.9 | 2.316 | 8.569 |
| 9 | -5.9 | 3.785 | 6.517 | 9 | -6.9 | 1.837 | 3.507 |
