# Supplementary material for: Discovering Thiamine Transporters as Targets of Chloroquine Using a Novel Functional Genomics Strategy
Source: PLoS Genet. 2012 Nov 29;8(11):e1003083. doi: 10.1371/journal.pgen.1003083 (PMC3510038; doi:10.1371/journal.pgen.1003083)
Supplement: Table S4 — Plasmids used in this study. (DOC) [file pgen.1003083.s010.doc]

| **Table S4. Plasmids used in this study.** | | |
| --- | --- | --- |
| **Plasmid** | **Genotype** | **Reference** |
| pRS416 | Vector, *CEN, URA3* | [43] |
| YEplac195 | Vector, *2µ, URA3* | [45] |
| pXP951 | *SLC19A3, 2µ, URA3*; *THI7* promoter and terminator | This study |
| pXP1033 | *thi7^R9G^, CEN, URA3* | This study |
| pXP1035 | *thi7^T287N^, CEN, URA3* | This study |
| pXP1037 | *thi7^E573G^, CEN, URA3* | This study |
| pXP1128 | *thi7^T287A^, CEN, URA3* | This study |
| pXP1164 | *thi7^T287D^, CEN, URA3* | This study |
| pXP1166 | *thi7^T287I^, CEN, URA3* | This study |
| pXP1168 | *thi7^T287Q^, CEN, URA3* | This study |
| ZHP96 | *THI3, 2µ, URA3* | This study |
| ZHP98 | *PDC2, 2µ, URA3* | This study |
| ZHP105 | *THI7* promoter and terminator*, CEN, URA3* | This study |
| ZHP106 | *THI7, CEN, URA3* | This study |
| ZHP107 | *thi7^R9G T287N E573G^, CEN, URA3* | This study |
| ZHP112 | *THI7, 2µ, URA3* | This study |
| ZHP113 | *NRT1, 2µ, URA3*; *THI7* promoter and terminator | This study |
| ZHP114 | *THI72, 2µ, URA3*; *THI7* promoter and terminator | This study |
| ZHP116 | *NEO1, 2µ, URA3* | This study |
